# Quality assurance for point-of-care testing in Mozambique’s National Health Service

**DOI:** 10.4102/ajlm.v5i2.445

**Published:** 2016-10-17

**Authors:** Patrina Chongo, Nádia Sitoe, Sofia Viegas, Isabel Pinto, Admiro Macave, Fernando Sitoe, Adolfo Vubil, Nédio Mabunda, Bindiya Meggi, Eduardo S. Gudo, Ilesh V. Jani

**Affiliations:** 1 National Institute of Health, Maputo, Mozambique; 2 National Medical Care, Ministry of Health, Maputo, Mozambique

## Health system and HIV epidemiology in Mozambique

Medical care in Mozambique is mostly provided through the national health service of the Ministry of Health. All primary healthcare and HIV-related services are provided free of charge. There are over 1500 public sector health facilities in Mozambique and most of these are primary healthcare centres. Although all hospitals have a laboratory, only a quarter of the health centres have a formal laboratory. In this context, point-of-care (POC) testing and syndromic management of diseases play an important role in the health system.

Both communicable and non-communicable diseases are prevalent in the Mozambican population. Mozambique has a population of 28 million and is among the nine countries with the highest HIV prevalence in the world.^1^ HIV prevalence in the country among people aged 15–49 years is 11.5%, ranging from 3.7% in the Niassa province in the north to 25.1% in the Gaza province in the south.^2,3^ HIV prevalence is higher among women (13.1%) than among men (9.2%), and higher in urban areas (15.9%) compared with rural areas (9.2%).^2,3^ Among children aged between 0 and 11 years, HIV prevalence is 1.4%, and 2.3% in those younger than one year.^2,3^ It is estimated that 102 new infections in children occur daily in Mozambique (Ministry of Health, unpublished data). Demographic impact studies show that an estimated 1.6 million Mozambicans were living with HIV in 2009.^2,3^

## Laboratory infrastructure and HIV-related testing in Mozambique

The Public Laboratory System encompasses 344 laboratories located in institutes, hospitals, health centres and non-governmental organisations. Most of these laboratories are directly managed by institutions affiliated with the Ministry of Health.

**FIGURE 1:**
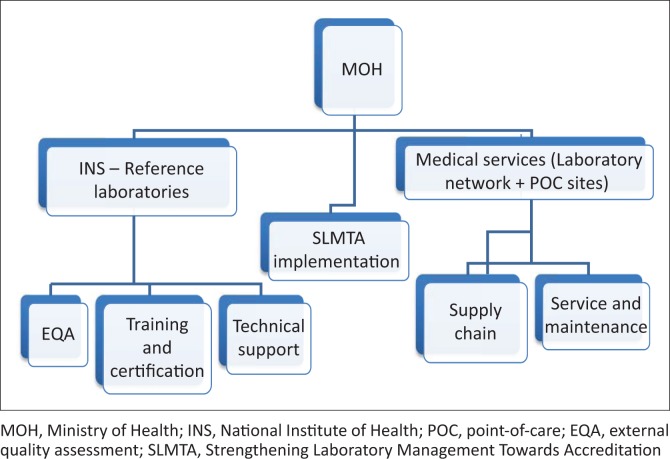
Governance structure of Mozambique’s public laboratory network.

Two branches within the Ministry of Health coordinate the laboratory network. Firstly, the Central Laboratory Department, which is part of the Directorate of Medical Services, is in charge of medical laboratories within all health facilities, assuring human resources, procurement of major equipment, consumables and reagents, and equipment maintenance (Figure 1). Secondly, the National Institute of Health (Instituto Nacional de Saúde; INS) houses the National Reference Laboratories and provides technical support to the laboratory network in terms of technology assessment, on-the job training, specialised testing, and external quality assessment.

HIV testing is performed in laboratories and at the POC within several departments of health facilities by using a serial algorithm of two rapid tests to detect antibodies against HIV. HIV testing is also widely used in voluntary counselling and testing facilities. From a handful of settings that tested for HIV in the late 1990s, today thousands of testing points exist nationwide. Annually, around six million HIV serological rapid assays are performed in Mozambique.^1^

CD4 T-cell counting in Mozambique was initially done using conventional laboratory-based flow cytometers. In 2003, three laboratories were providing CD4 testing for the whole country. Currently, 201 CD4 testing devices exist in the country. Of these, 45 are laboratory-based technologies, 19 are full flow cytometers and 26 are dedicated bench-top cytometers. The remaining 156 instruments are of the POC type and are placed in various settings within and outside laboratories, including 13 located in mobile clinics. Annually, around 250 000 CD4 T-cell enumerations are performed in Mozambique (CD4 EQA INS data).

Early infant diagnosis (EID) was first performed in Mozambique, using manual PCR assays, at the INS reference laboratory in 2005. Currently, EID takes place in five laboratories, one in the north, two in the centre and two in the south of the country, using high-throughput PCR methods on dried blood-spot specimens. Collection of dried blood-spots is done in 1136 health facilities nationwide, and collected samples are sent to one of the five network laboratories. Results of testing are sent back mainly using mobile phone technology to printers located at the health facility level (www.portaldpi.com). Annually, 60 000 exposed children undergo EID testing in Mozambique, and viral load testing for treatment monitoring is currently being established within EID laboratories.^1^ POC technologies for EID and viral load testing are under evaluation, with implementation planned during 2016.

## Framework for quality assurance of diagnostics in Mozambique

A National Laboratory Policy document has been drafted and awaits formal approval by the government. This Policy sets clear responsibilities related to quality assurance at the different levels of the health system. Currently, quality assurance activities in Mozambique include:

**Strengthening internal management and internal quality control:** This is done mainly through the implementation of the Strengthening Laboratory Management Towards Accreditation (SLMTA) tool.^4,5,6^ Since its inception in Mozambique in 2011, 32 laboratories have benefited from this programme.

**FIGURE 2:**
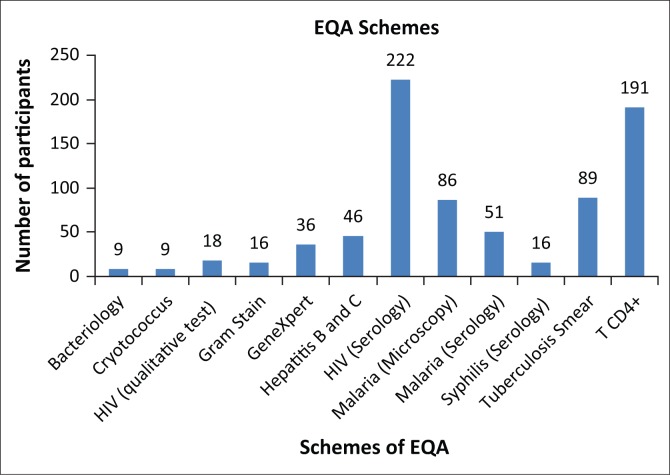
Schemes for external quality assessment (EQA) in Mozambique, 2016.

**External Quality Assessment (EQA):** The EQA programme in Mozambique started in pilot form, sending out proficiency panels for CD4 T-cell enumeration in 2005. Currently, the EQA programme covers 451 sites and includes: (i) Proficiency panels for 12 schemes (Figure 2), which, with the exception of CD4 counting, GeneXpert^®^ and bacteriology, are prepared in Mozambique. For CD4 counting, INS and QASI (Public Health Agency of Canada) are planning a training activity to build national capacity for proficiency panel preparation; (ii) blind re-checking for tuberculosis smear and malaria smear microscopy; and (iii) data analysis collected from CD4 counting POC devices using mobile phone technology. This activity is done in addition to the proficiency testing described in (i) above.

Site visits are carried out at all sites that have low performance on two consecutive panels. Follow-up visits may be carried out to monitor implementation of corrective measures.

Regulatory approval systems for new diagnostics are currently being established and designed based on existing platforms for drugs and vaccines. Most current policies do not differentiate laboratory-based from POC technologies.

## Lessons learnt from existing quality assurance programmes

### SLMTA tool implementation

The following lessons were learned from implementing SLMTA in Mozambique:

- Encouraging the entire laboratory to work as a team has guaranteed better results and increased confidence in the programme.
- Reporting SLMTA activities to the hospital management has motivated local leaders to monitor the implementation of quality systems in clinical laboratories.
- Implementation of the SLMTA tool has shown better results when done together with mentorship from nationally-recognised leaders.
- Accreditation at the highest standards is possible for public medical laboratories in Africa, even under challenging environments. In 2015, the National Tuberculosis Reference Laboratory of the INS received ISO 15189 accreditation by the Instituto Português para Acreditação for fluorescence smear microscopy and for culture in liquid and solid media assays.

### External quality assessment

The release of a periodic overall report, where sites are not identifiable, but which shows the performance of the laboratory network, is important with regard to creating awareness about the quality of diagnostic testing. Presentation of annual certificates to participating sites increases confidence in the programme and boosts the willingness of sites to participate in subsequent rounds. In addition, EQA results improve the confidence of results that are produced by POC tests and allow the identification of sites and operators with performance problems. Site visits positively impact the performance of proficiency testing, and strategies to establish the capacity to produce proficiency panels at the national level have allowed the country to increase the coverage and efficiency of EQA programmes. A further observation is that innovations, such as the use of mobile phone connectivity on diagnostic platforms, are critical for assuring the quality of the next generation of POC assays.

## National quality assurance programme for point-of-care testing

The laboratory network in Mozambique is a hybrid and dynamic environment where both laboratory-based and POC assays co-exist with complementary roles. Therefore, in this context, and given the paucity of resources, the quality of POC testing is not the target of a distinct programme. Current quality assurance activities cover both laboratory-based and POC assays. Nevertheless, specific operational features of POC tests demand focused solutions within the programme. For example, training of lay counsellors for HIV testing and of nurses for POC CD4 testing has required the establishment of specific training packages and appropriate supervision checklists.

Mozambique developed a guideline for a POC implementation, where participation in an EQA programme is mandatory and must be part of the process. With the rapid increase in the implementation of technology, specifically for CD4 testing, the EQA provider faced problems with fulfilling all sites and is still facing problems adding more sites. Based on that, it has been challenging for the programme to start preparing in-country panels to cover these sites.

The main EQA activities in place in Mozambique include managing proficiency testing panels, building capacity for producing panels in-country, and performing corrective actions (Table 1).

**TABLE 1:** Mozambique external quality assessment activities in place, 2016.

EQA activity	CD4	EID	Viral Load
Management of panels	In-country coordinator (114 sites)	In-country coordinator (4 sites)	1 site
Coverage	76% (86 sites out)	100%	100%
Providers	QASI	NICD	CDC
Capacity to build panels in-country	Yes	Yes	Yes
Corrective action performed	Yes	Yes	Yes

EID, early infant diagnosis; VL, viral load; QASI, Public Health Agency of Canada; NICD, National Institute for Communicable Diseases; CDC, US Centers for Disease Control and Prevention.

## The way forward

The health system is constantly subjected to external forces such as demographic changes, epidemiological transitions, scientific discoveries, technological innovations and public health breakthroughs. The demands for POC testing to improve access will continue to increase, thus the capacity to produce proficiency panels in Africa will allow quality assurance programmes to keep up with the demand of POC testing. The laboratory network and associated quality assurance programmes must remain in readiness for the challenges facing our continent.
